# Effects of cultivation duration on soil organic carbon components, bacterial communities, metabolites and bamboo growth in the *Phyllostachys edulis*– *Ganoderma lucidum* agroforestry systems

**DOI:** 10.3389/fpls.2026.1917212

**Published:** 2026-07-30

**Authors:** Huijing Ni, Haoyu Chu, Bo Wang

**Affiliations:** 1 Zhejiang Academy of Forestry, Northwest Zhejiang Bamboo Forest Ecosystem Positioning Observation and Research Station, Hangzhou, China; 2 International Center for Bamboo and Rattan Sanya Research Base, Sanya, China

**Keywords:** bacterial communities, bamboo aboveground biomass, metabolites, *Phyllostachys edulis–Ganoderma lucidum* agroforestry systems, soil organic carbon components

## Abstract

Agroforestry systems are a production mode in bamboo forest management that balance ecological and economic benefits. Soil carbon (C) pools form the most important and stable C sinks. However, there is limited information on soil C pool components, bacterial communities, metabolites and bamboo growth in agroforestry systems. Here, *Phyllostachys edulis*–*Ganoderma lucidum* agroforestry systems with cultivation durations of 1, 2, and 3 years were selected, and pure Moso bamboo forest as the control (CK). We examined the variation in soil organic C components, related enzyme activities, bacterial community structure and diversity, metabolites and bamboo aboveground biomass in response to different cultivation years of *G. lucidum*, and explored their interrelationships. The results showed that cultivation of *G. lucidum* significantly enhanced bamboo aboveground biomass by 30.2%–40.2%, and concurrently increased soil total organic carbon (SOC) and mineral-associated organic carbon (MOC) by 14.23%–34.73% and 27.13%–120.71%, respectively, along with the extension of cultivation duration. Cultivation of *G. lucidum* for more than 2 years enhanced soil catalase activity, bacterial community diversity and richness, and the relative abundances of Proteobacteria, Actinobacteriota, and Verrucomicrobiota. Six significantly upregulated differential metabolites were shared between different cultivation durations and belonged to the categories of amino acids and their derivatives, organic acids, and benzene and its derivatives. Structural equation modeling analysis revealed that cultivation durations exerted a direct positive impact on changes in the soil bacterial Actinobacteriota, Verrucomicrobiota, and indirect positive impact on MOC, thereby indirectly promoting bamboo aboveground biomass. In conclusion, agroforestry promotes soil C and bamboo productivity, providing a theoretical basis for enhancing bamboo biomass through the targeted regulation of soil bacterial and metabolite profiles, thus supporting the sustainable management of Moso bamboo forests.

## 1 Introduction

Moso bamboo (*Phyllostachys edulis*) is a bamboo species with a large cultivation area in China. The largest and most economically valuable bamboo species reproduce rapidly and have a short maturity period for bamboo stems. This allows sustainable use from a single plantation (Ni and Su, 2024). Under traditional management practices, bamboo forest products, primarily bamboo timber and bamboo shoots, are relatively limited. Their production has already reached a high level, making it difficult to increase further over a short period of time (Yang et al., 2019). Agroforestry is a land use system in which trees are managed with crops and/or animal production systems in agricultural settings. This includes planting trees in agricultural systems and cropping and/or feeding animals in forest ecosystems (Li et al., 2022). Composite management can fully use the understory space and provide several sustainable effects (Ni et al., 2025; Wang et al., 2023; Yang et al., 2020). The integrated management of bamboo mushrooms is a vital practice in integrated bamboo forest management. Bamboo forests create favorable habitats for edible mushroom growth, meanwhile, mushroom residues function as organic fertilizers for bamboo stands, forming a synergistic system of bamboo with mushrooms and mushrooms promoting bamboo growth (Ye et al., 2026; Wang et al., 2026). *Ganoderma lucidum* (*G. lucidum*), a medicinal fungus belonging to the family Ganodermataceae and genus Ganoderma, is a renowned and valuable traditional Chinese medicine. Its cultivation is currently concentrated in greenhouses and indoor facilities, with relatively few wild cultivation practices. The shaded and humid microenvironment of bamboo forests provides ideal conditions for *G. lucidum* cultivation, and successful intercropping practices have been well established in previous studies.

Soil is a carrier of material and energy exchange in forest ecosystems, providing essential water and nutrients for plant growth (Li et al., 2023). Soil quality is a manifestation of soil physical, chemical, and biological properties. It is the most sensitive indicator of soil dynamic changes, playing an important role in maintaining long-term forest productivity (Yang et al., 2025). SOC is the largest terrestrial carbon (C) pool in the tropics and is a key component of soil that affects its physical, chemical, and biological properties (Adiyah et al., 2023). Soil C sequestration depends on complex interactions among biological, chemical, and physical processes that respond differently to different environmental conditions (Pan et al., 2025). Understory intercropping has been widely recognized as an effective agroforestry practice for improving soil quality and ecosystem functions (Cherubin et al., 2019; Huang et al., 2022; Hübner et al., 2021; Yi et al., 2022; Baah-Acheamfour et al., 2014).

Experiments on planting medicinal plants in a temperate forest understory have found that understory intercropping substantially improves soil nitrogen (N) and phosphorus (P) availability and enhances enzyme activities, such as urease and phosphatase (Ma et al., 2017; Richards et al., 2010). Long-term trials of understory cropping, such as ginger and tea in Southeast Asian rubber plantations have shown that the intercropping system increases soil organic matter (SOM) content by 15–30%, improve soil water retention capacity, and reduce surface runoff (Chen et al., 2019). For instance, intercropping with leguminous plants substantially increases the abundance of nitrogen-fixing bacteria, enhances soil β-glucosidase activity, and promotes C cycling (Cong et al., 2015). In degraded forested lands, intercropping with perennial understory crops can restore soil fertility, reduce the risk of heavy metal migration, and enhance the stability of soil aggregates (Isaac et al., 2019). These findings indicate that the ecological outcomes of understory intercropping depend largely on the functional traits of the intercropped species. Among various understory cropping systems, edible mushroom cultivation has attracted particular attention due to its dual benefits in generating economic returns and influencing soil biogeochemical cycles. The cultivation of edible mushrooms such as shiitake and oyster mushrooms in forest stands has been shown to affect soil C and N cycling. Moreover, returning spent mushroom substrate to the field considerably increased soil organic matter by 20–35% and the available of N and P, while enhancing the abundance of ectomycorrhizal fungi, which promotes nutrient uptake by forest trees (Li et al., 2021). In bamboo forest systems, the trial of intercropping shiitake mushrooms in bamboo forests indicated a short-term decrease in soil bacterial diversity but a significant increase in fungal abundance, particularly that of saprophytic fungi. The mycelial network enhances soil aggregate formation, and soil pH stabilizes after the degradation of the spent mushroom substrate (Nakatsuka et al., 2016).

Previous studies have shown that agroforestry management can affect soil organic C by influencing soil bacteria and metabolites. A study found that planting *Panax notoginseng* significantly increased the content of eight differentially accumulated metabolites (DAMs) in the soil of *Pinus armandii*, and structural equation modeling showed that soil enzymes, metabolites, and soil factors jointly enhanced the complexity of microbial networks (Hei et al., 2025). Another research has found that the content of metabolites (such as columbin and glycyl-L-leucine) in rhizosphere soil significantly changes under understory planting mode, and is significantly correlated with rhizosphere microbial diversity and community complexity (Wang et al., 2025). The synergistic interaction between bacterial communities changes the utilization strategy of microorganisms for glucose and SOC, leading to sustained inhibition of SOC mineralization (Hu et al., 2026). These findings all elucidate the pathway relationships between bacterial communities, metabolites, and soil C.

Nevertheless, changes in soil organic carbon, bacterial communities, metabolites, and their relationships have been limited to *P. edulis*–*G. lucidum* agroforestry system. In this study, we aimed to explore (1) how cultivation duration affects changes in soil organic carbon components, bacterial communities, and metabolites in *P. edulis*–*G. lucidum* agroforestry systems, and (2) the internal links among soil organic carbon components, bacterial communities, metabolites and bamboo aboveground biomass.

## 2 Materials and methods

### 2.1 Site description and experimental design

The site is located in Deqing county, Huzhou city, Zhejiang Province, China (30°26′−30°42′ N, 119°45′−120°21′ E). It has a northern subtropical monsoon climate, with an average annual temperature of 13−16 °C, a frost-free period of 220−236 d, and an average annual precipitation of 1379 mm. Moso bamboo forests have been cultivated for over 30 years, with extensive management and harvesting of bamboo trunks and shoots every two years. The diameter at breast height (DBH) and stand density of the forests were 9.6−11.2 cm and 2520−2775 stems/ha. The soil bulk density and pH were 1.02−1.18 g/cm^3^ and 5.14−5.42, respectively.

*G. lucidum* was cultivated and planted in January 2021, 2022, and 2023. We cleaned up the mixed irrigation under the forest, dug holes horizontally along the contour line, with a spacing of 1 m between holes and a spacing of 1.5 m between the horizontal bands. The mushroom sticks were then inserted into the holes, covered with soil, and watered from the top of each mushroom stick. The mushrooms started to grow in June, its edge was sealed in July, before being covered with spore powder and *G. lucidum*. After harvesting, the remaining mushroom sticks rotted and decomposed. Meanwhile, the bamboo was cut every two years to maintain a consistent bamboo forest density.

### 2.2 Soil sampling

The method of space-for-time substitution, an effective way to study changes over time (Sparling et al., 2003), was used to monitor soil changes occurring along an agroecosystem at 1 (A1), 2 (A2), and 3 (A3) years of cultivation duration, with pure Moso bamboo forest as the control (CK). Agroecosystem plantations established at different times provide an ideal opportunity to study the *G. lucidum* planting process, as soil conditions prior to planting are largely shaped by geomorphologic processes.

A total of 24 soil samples (0–30 cm depth) were collected from four plots in May 2024, with sampling locations evenly interspersed among bamboo plants and mushroom sticks. The fresh soil samples were passed through a 2 mm sieve. The soil sample were divided into three parts, one part naturally air dried, one part stored in a refrigerator at 4 °C, and one part stored in a refrigerator at −80 °C, respectively.

### 2.3 Determination of soil basic properties, carbon components forms and enzyme activities

Soil pH was measured using the potentiometric method with a soil-to-water ratio of 1:2.5. The total nitrogen (TN) was determined using the Kjeldahl method after sulfuric acid digestion. Alkali-hydrolyzable nitrogen (alkali-N) was measured using the alkali hydrolysis diffusion method. Total phosphorus (TP) was analyzed using the NaOH fusion–molybdenum antimony anti-spectrophotometric method. Available phosphorus (AP) was determined using the NH_4_F–HCl extraction method. The cation exchange capacity (CEC) was measured using cobalt hexamine trichloride extraction followed by spectrophotometric analysis.

SOC was determined by the external heating potassium dichromate oxidation method. Particulate organic carbon (POC) and mineral-associated organic carbon (MOC) were extracted using sodium hexametaphosphate and measured using the external heating potassium dichromate oxidation method. Soil enzyme activities, including β-1,4-glucosidase (BG), catalase (CAT), and polyphenol oxidase (PPO), were measured using commercial assay kits.

### 2.4 DNA extraction, high-throughput sequencing, and bioinformatics

Soil DNA was extracted from a 0.5 g soil sample using a soil DNA Kit (Magen, Guangzhou, China) according to the manufacturer’s protocols. The V3-V4 region was amplified using the following primers: 5′-CCTACGGGNGGCWGCAG-3′ (338F) and 5′-GACTACHVGGGTATCTAATCC-3′ (806R).

The 16S rDNA target region of the ribosomal RNA gene was amplified using PCR (95°C for 5 min, followed by 30 cycles at 95°C for 1 min, 60°C for 1 min, and 72°C for 1 min and a final extension at 72°C for 7 min). 50 µL mixture containing 10 µL of 5 × Q5@ Reaction Buffer, 10 µL of 5 × Q5@ High GC Enhancer, 1.5 µL of 2.5 mM dNTPs, 1.5 µL of each primer (10 µM), 0.2 µL of Q5@ High-Fidelity DNA Polymerase, and 50 ng of template DNA. PCR reagents were purchased from New England Biolabs (USA). The PCR-purified products from each locus were mixed and sequenced using Illumina on a NovaSeq 6000 platform at Gene Denovo (Guangzhou, China).

After the sequencing data was downloaded, the Amplicon Sequence Variants (ASVs) feature list was generated using the DADA2 (Divisive Amplicon Denoising Algorithm 2) pipeline (Yang et al., 2025). Taxonomic annotations were assigned to the ASVs using the SILVA taxonomy database (Yang et al., 2025). Further analyses were conducted using the ASV abundance table after normalization to account for variations in sequencing depth across samples.

### 2.5 LC–MS-based untargeted metabolomics analysis

250 mg (± 1 mg) of the sample was weighed into the centrifuge tube and 500 uL of −20 °C precooled 70% methanol water internal standard extraction solution was added to the sample. The samples were vortexed for 3 min and ultrasonicated in an ice-water bath for 10 min. The samples were then removed and vortexed for 1 min. They were then left to stand in a −20 °C refrigerator for 30 min. Under 4 °C conditions, they were centrifuged at 12,000 r/min for 10 min. All the supernatant was transferred and put through a 0.22 μm filter before being injected into the LC–MS/MS system for analysis. One aliquot was analyzed using positive ion conditions and was eluted from the T3 column (Waters ACQUITY Premier HSS T3 Column 1.8 µm, 2.1 mm * 100 mm) using 0.1% formic acid in water as solvent A and 0.1% formic acid in acetonitrile as solvent B in the following gradient: 5 to 20% in 2 min. It was then increased to 60% in the following 3 mins, increased to 99% in 1 min and held for 1.5 min, before being brought back to 5% mobile phase B within 0.1 min and held for 2.4 min. The analytical conditions were as follows: column temperature, 40°C; flow rate, 0.4 mL/min; injection volume, 4 μL. Another aliquot was analyzed using negative ion conditions and was the same as the elution gradient of the positive mode.

Data acquisition was performed in information-dependent acquisition (IDA) mode using Analyst TF 1.7.1 Software (Sciex, Concord, ON, Canada). The source parameters were set as follows: ion source gas 1 (GAS1), 50 psi; ion source gas 2 (GAS2), 50 psi; curtain gas (CUR), 25 psi; temperature (TEM), 550°C; declustering potential (DP), 60 V, or −60 V in positive or negative modes, respectively; and ion spray voltage floating (ISVF), 5000 V or −4000 V in positive or negative modes, respectively. The TOF MS scan parameters were set as follows: mass range, 50–1000 Da; accumulation time, 200 ms; and dynamic background subtraction, ON. The product ion scan parameters were set as follows: mass range, 25–1000 Da; accumulation time, 40 ms; collision energy, 30 or −30 V in positive or negative modes, respectively; collision energy spread, 15; resolution, UNIT; charge state, 1 to 1; intensity, 100 cps; exclude isotopes within 4 Da; mass tolerance, 50 ppm; maximum number of candidate ions to monitor per cycle, 18.

### 2.6 Moso bamboo aboveground biomass production

Moso bamboo aboveground biomass in each plot was estimated with the equation (Ni et al., 2021)

Moso bamboo aboveground biomass (kg/hm^2^)


$$\text{M}=747.784^{2.771}\text{D}{\left[\left(0.148\times \text{A}\right)/\left(0.028+\text{A}\right)\right]}^{5.555}+3.772$$

where M is aboveground biomass of an individual bamboo (kg); D is the DBH of each bamboo (cm); A is the age of bamboo (y).

### 2.7 Statistical analysis

All the statistical analyses were performed using SPSS 20.0. and Excel 2016. One-way analysis of variance followed by Duncan’s multiple range test was used to detect significant differences (*p<* 0.05) between treatment means. To evaluate the relationships between the bacterial community structure and environmental variables, the first axis of the NMDS analysis was extracted and used as a proxy variable representing the community structure in subsequent Pearson correlation and regression analyses. Pearson’s correlation and redundancy analyses were conducted to examine the links between the soil carbon component forms, enzymatic activities, and bacterial communities.

Partial least squares structural equation modeling (PLS-SEM) was conducted to examine the relationships between basic soil properties, carbon components, enzymatic activities, bacterial diversity, metabolites and bamboo aboveground biomass. Data from CK, A1, A2, and A3 were used to construct the PLS-SEM with plspm package of R software. All the figures were created using Origin 2024 software and the R package.

## 3 Results

### 3.1 Moso bamboo aboveground biomass

**Figure 1:**
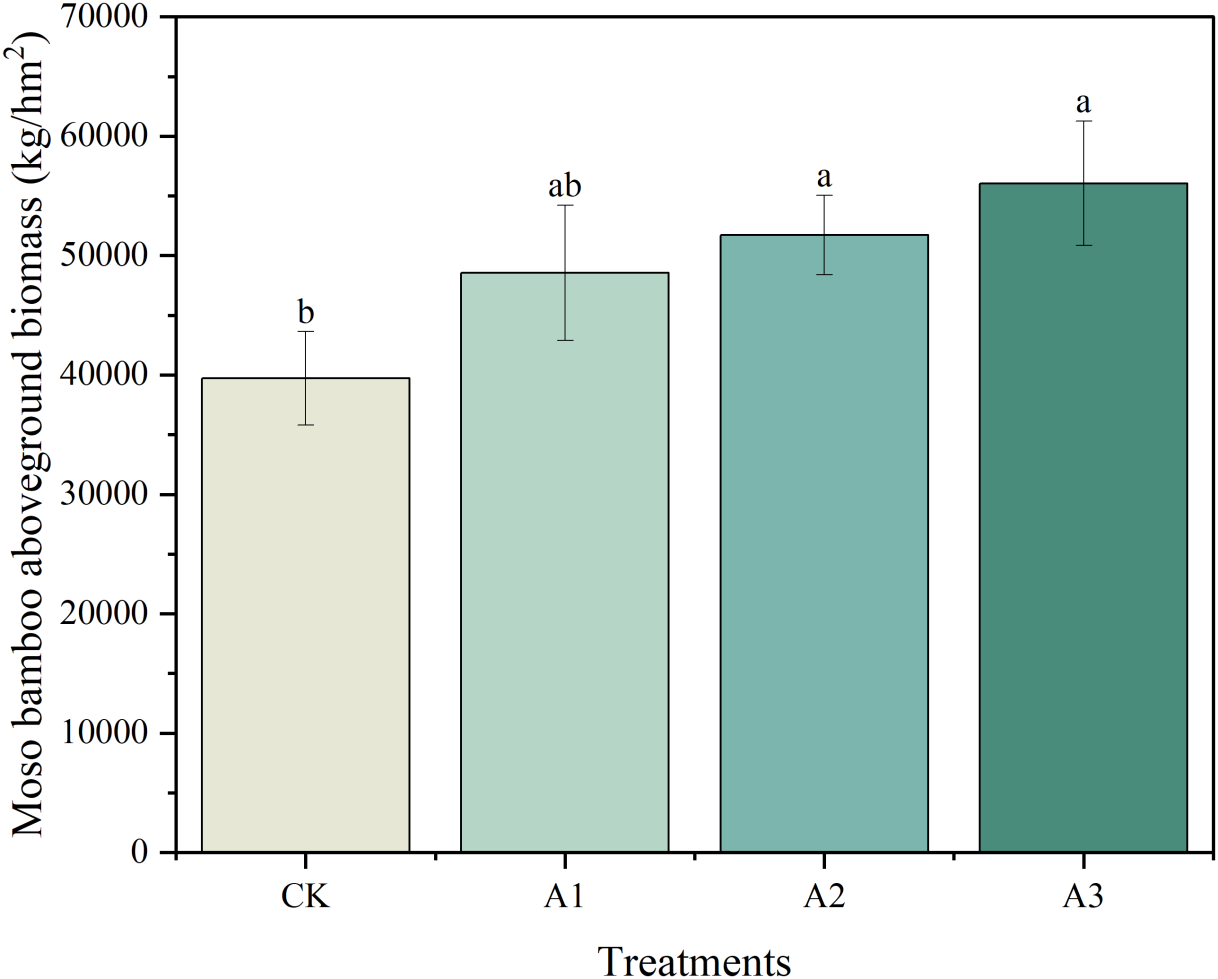
Moso bamboo aboveground biomass in *P. edulis*–*G. lucidum* agroforestry models.

The Moso bamboo aboveground biomass was significantly affected by cultivation duration in *P. edulis–G. lucidum* agroforestry systems. The A2 and A3 treatments increased the aboveground biomass by 30.2% and 40.2%, respectively (**Figure 1**).

### 3.2 Soil basic properties

As shown in **Table 1**, the cultivation of *G. lucidum* significantly reduced soil pH in Moso bamboo plantations, with decreases of 4.39%, 2.26%, and 3.34% observed in the A1, A2, and A3 treatments, respectively. Compared with the CK, A1 had no significant effect on soil CEC. Meanwhile, CEC significantly decreased in A2 and A3 by 18.16% and 26.49%, respectively. The A2 and A3 treatments significantly increased soil TN by 8.66% and 33.07%, respectively. Only A3 significantly enhanced TP content, with an increase of 29.63%. The AP content in A1 was significantly higher than in the control, with an increase of 38.94%. Meanwhile, AP in A2 and A3 were significantly lower than in the control, decreasing by 43.75% and 11.54%, respectively. The cultivation of *G. lucidum* altered the soil properties of Moso bamboo plantations, and these changes were associated with the duration of *G. lucidum* cultivation.

**Table 1:** Soil basic properties in *P. edulis*–*G. lucidum* agroforestry models.

Treatments	pH	CEC	TN	TP	AN	AP
		cmol/kg	g/kg	g/kg	mg/kg	mg/kg
CK	5.39 ± 0.03a	6.03 ± 0.57a	1.27 ± 0.04c	0.27 ± 0.03b	156.12 ± 0.38c	2.08 ± 0.04b
A1	5.15 ± 0.02d	6.27 ± 0.19a	1.26 ± 0.04c	0.24 ± 0.01b	145.86 ± 0.96d	2.89 ± 0.08a
A2	5.27 ± 0.02b	4.93 ± 0.34b	1.38 ± 0.02b	0.26 ± 0.01b	159.05 ± 1.76b	1.17 ± 0.04d
A3	5.21 ± 0.01c	4.43 ± 0.25b	1.69 ± 0.02a	0.35 ± 0.02a	173.30 ± 1.50a	1.84 ± 0.08c

Values represent the average of six replicates ± standard deviations. Different lowercase letters indicate significant differences (*p* < 0.05) between treatments.

### 3.3 Soil carbon components and content

**Figure 2:**
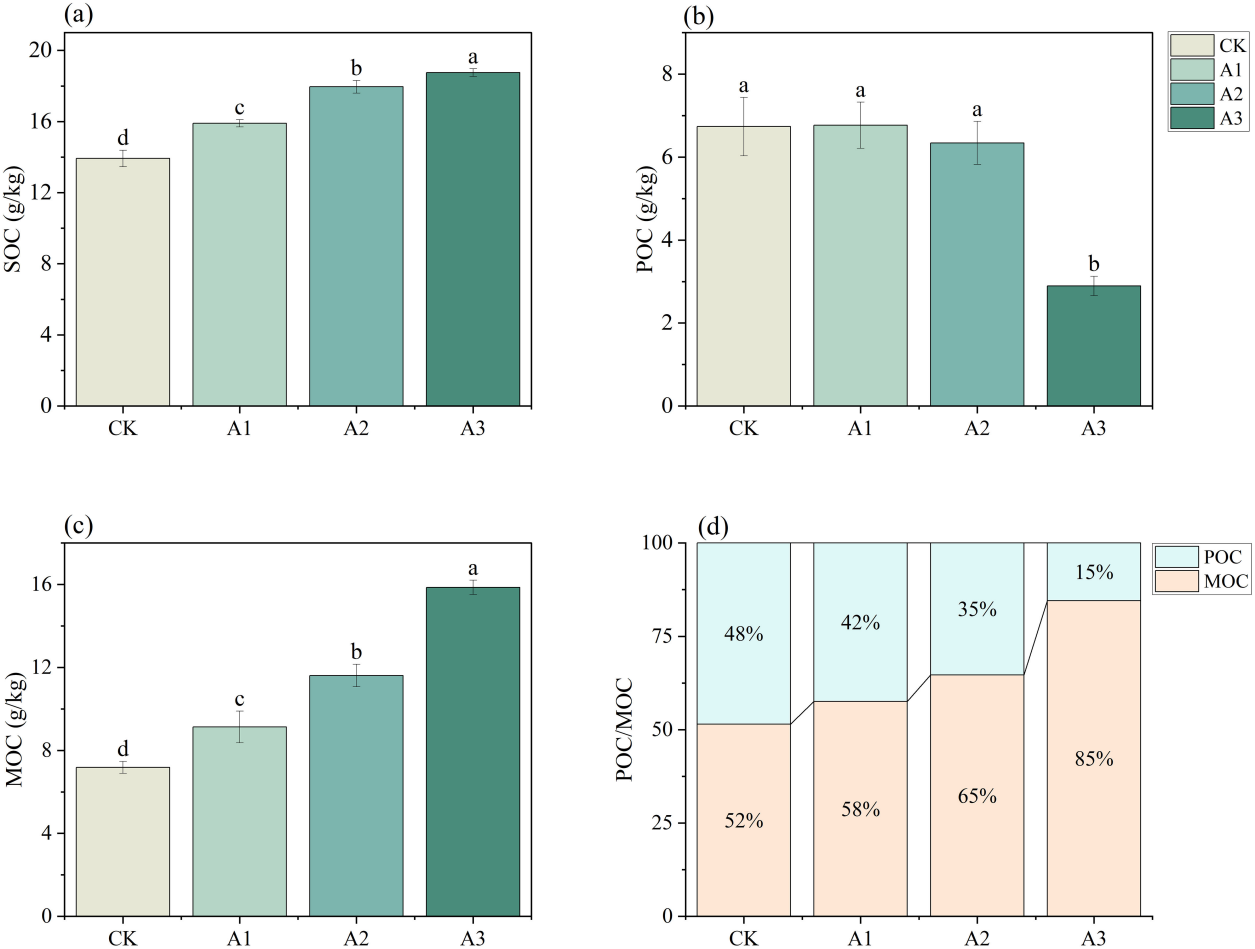
Soil carbon components and content in *P. edulis*–*G. lucidum* agroforestry models. **(A)** SOC. **(B)** POC. **(C)** MOC. **(D)** POC/MOC. Different lowercase letters indicate significant differences (*p* < 0.05) between treatments. Error bars indicate standard deviations. The same below.

*G. lucidum* cultivation significantly altered the contents of SOC, POC, and MOC in *P. edulis*–*G. lucidum* agroforestry models. These changes were strongly influenced by cultivation duration (**Figure 2**). Compared with the control, *G. lucidum* cultivation significantly increased SOC content by 14.23% to 34.73%. This showed a consistent upward trend with increasing cultivation time, following the order A3 > A2 > A1 > CK (**Figure 2A**). After three years of cultivation, POC content decreased significantly by 57.02%. Meanwhile, no significant differences were observed between A1, A2, and CK. In contrast, the MOC content was significantly elevated in all *G. lucidum* treatments, increasing from 27.13% to 120.71%. It also showed a clear accumulation trend with longer cultivation durations. In the CK treatment, the proportions of POC and MOC were nearly balanced, accounting for 48% and 52% of SOC, respectively. However, in the A3 treatment, MOC increased to 85%, whereas POC declined to 15%.

### 3.4 Soil enzyme activities

**Figure 3:**
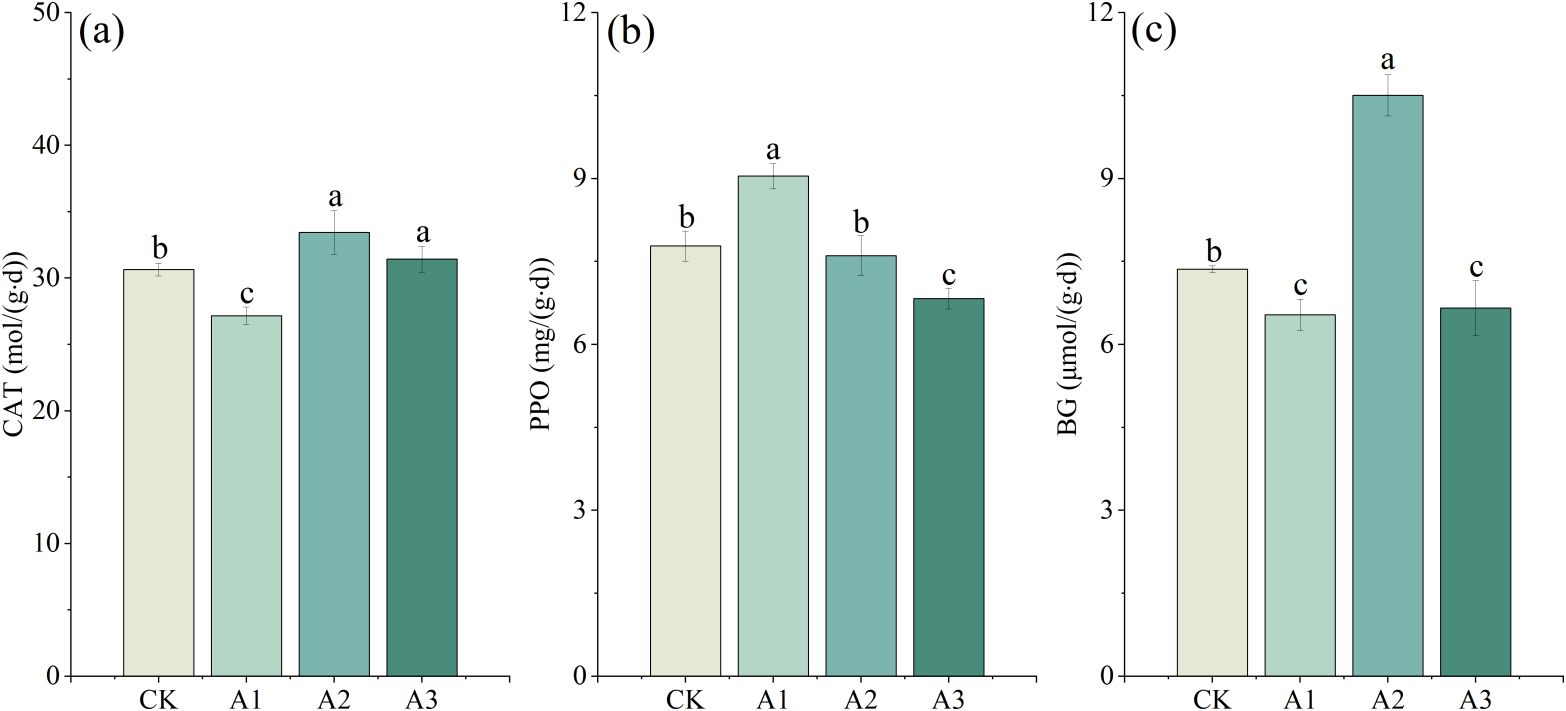
Soil enzyme activities in *P. edulis*–*G. lucidum* agroforestry models. **(A)** CAT. **(B)** PPO. **(C)** BG.

**Figure 3** illustrates the changes in soil enzyme activities related to C cycling under *P. edulis*–*G. lucidum* agroforestry models with different cultivation durations. Compared to CK, the A1 treatment reduced CAT activity by 11.39%, whereas A2 and A3 treatments increased CAT activity by 9.10% and 2.51%, respectively. The A1 and A3 treatments significantly affected PPO activity, with A1 significantly increasing it by 16.32% and A3 significantly decreasing it by 12.21%. A2 significantly enhanced BG activity, whereas A1 and A3 significantly suppressed it.

### 3.5 Soil bacterial community structures

**Figure 4:**
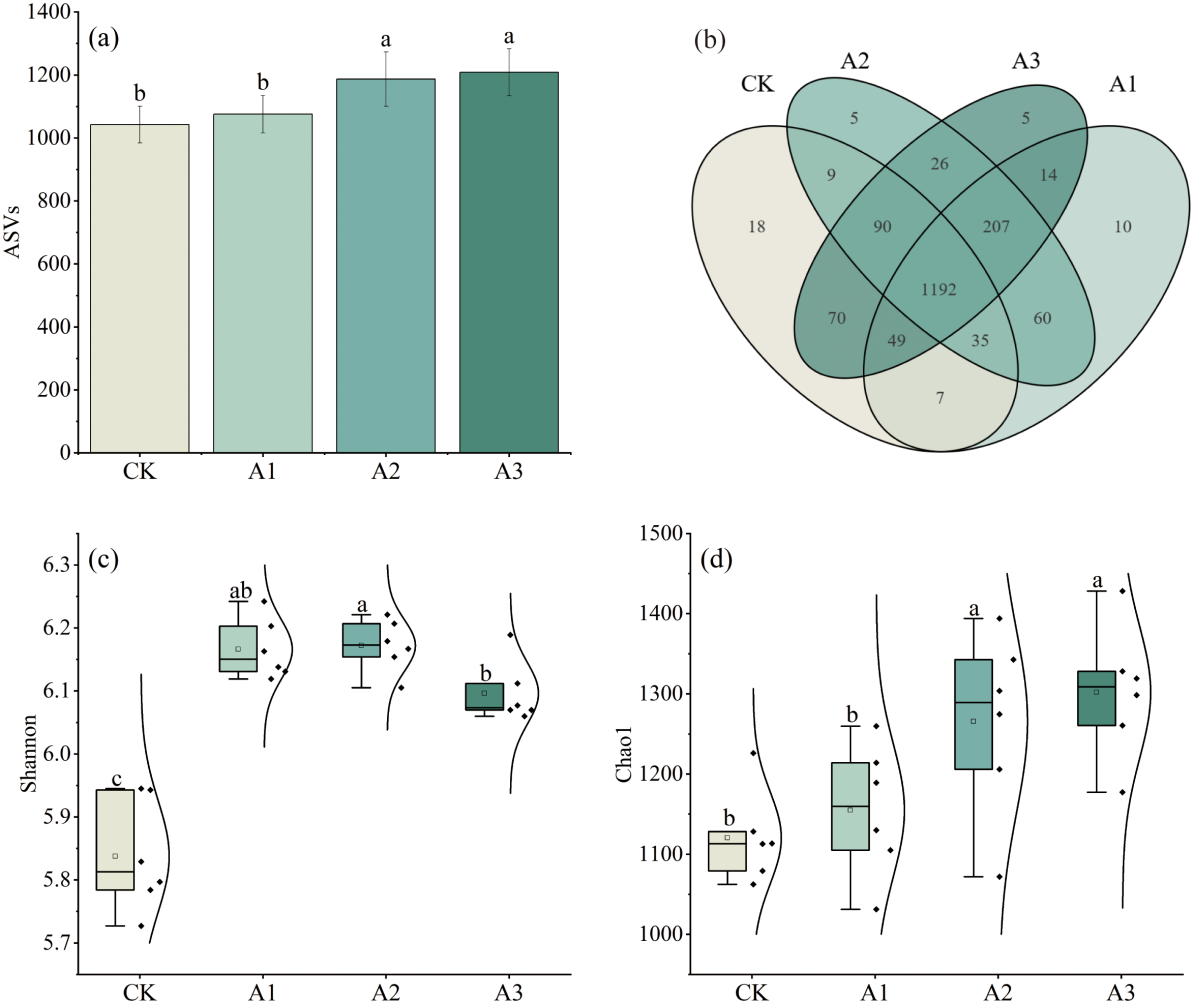
Sequence statistics and alpha of soil bacteria in *P. edulis*–*G. lucidum* agroforestry models. **(A)** ASVs counts of soil bacteria. **(B)** Venn diagram of soil bacteria community. **(C)** Shannon index. **(D)** Chao 1 index.

The cultivation of *G. lucidum* significantly altered the diversity and structure of soil bacterial communities in Moso bamboo plantations. These changes were influenced by cultivation duration. As shown in **Figure 4A**, A2 and A3 treatments significantly increased the number of bacterial ASVs by 13.89% and 15.96%, respectively, compared to the control. The Venn diagram showed shared and unique ASVs among treatments (**Figure 4B**). In total, 1,192 ASVs were shared across all four treatments, whereas CK, A1, A2, and A3 contained 18, 10, 5, and 14 unique ASVs, respectively. Therefore, the treatments influenced the formation of specific bacterial taxa. The Shannon index, which reflects microbial diversity and evenness, was significantly increased by *G. lucidum* cultivation, with increases ranging from 4.46% to 5.83% (**Figure 4C**). However, the Shannon index showed a decreasing trend with longer cultivation duration. An estimator of ASV richness, the Chao1 index was also significantly elevated under *G. lucidum* cultivation (**Figure 4D**). Its trend closely matched that of the ASV counts, further supporting the idea that the A2 and A3 treatments promoted a higher potential species richness in the bacterial community.

**Figure 5:**
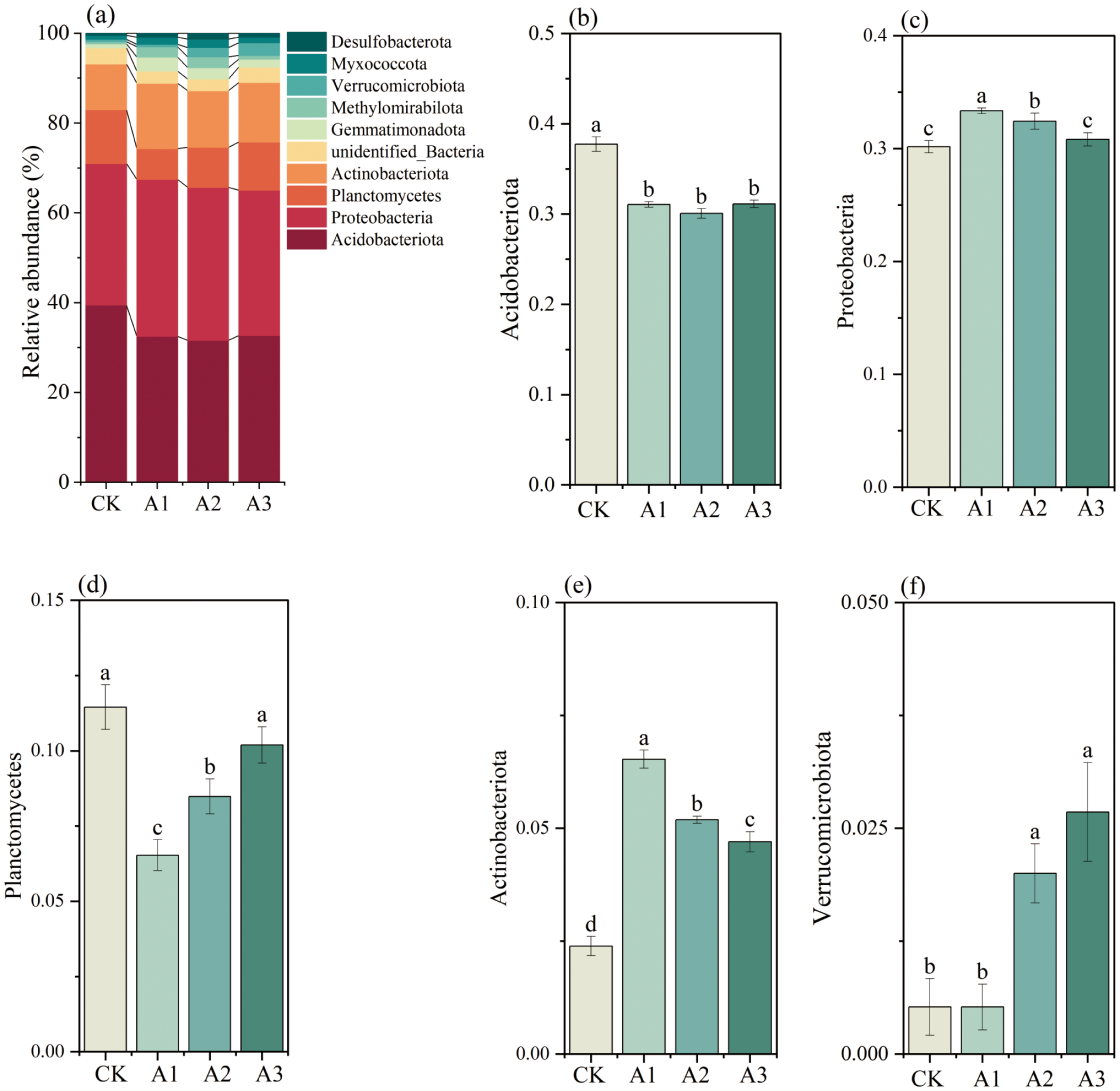
Relative abundance and **(A)** Relative abundance of soil bacteria at the phylum level. **(B–F)** Relative abundance of Acidobacteriota, Proteobacteria, Planctomycetes, Actinobacteriota, Verrucomicrobiota. Phylum-Level differences of soil bacteria in *P. edulis*–*G. lucidum* agroforestry models ().

**Figure 5** shows the changes in the relative abundance of soil bacterial communities at the phylum level in *P. edulis*–*G. lucidum* agroforestry models with different cultivation durations. At the phylum level (**Figure 5A**), Acidobacteriota, Proteobacteria, Planctomycetes, and Actinobacteriota were predominant at the dominant phylum. Cultivation of *G.lucidum* significantly decreased the relative abundance of Acidobacteriota and Planctomycetes(**Figures 5B, D**). Meanwhile, it increased that of Proteobacteria, Actinobacteriota, and Verrucomicrobiota (**Figures 5C, E, F**). To further clarify the effects of cultivation duration on the soil microbial community structure, NMDS and LEfSe analyses were performed (**Supplementary Figure 1**). The NMDS plot (**Supplementary Figure 1A**) showed a clear separation among the four treatment groups (CK, A1, A2, and A3) along the two principal axes with a stress value of 0.047, indicating dimensionality reduction and significant differences in microbial community structures among treatments. The CK group was separated from the three *G. lucidum* cultivation treatments. This suggests that the *P. edulis*–*G. lucidum* agroforestry model significantly altered soil bacterial community structure. Treatments A1, A2, and A3 formed distinct, independent bacterial community clusters. LEfSe analysis further demonstrated that the dominant bacterial taxa varied significantly across treatments. The number of dominant taxa decreased with *G. lucidum* cultivation and showed a decreasing trend with increasing cultivation duration.

### 3.6 Soil metabolite characteristics

**Figure 6:**
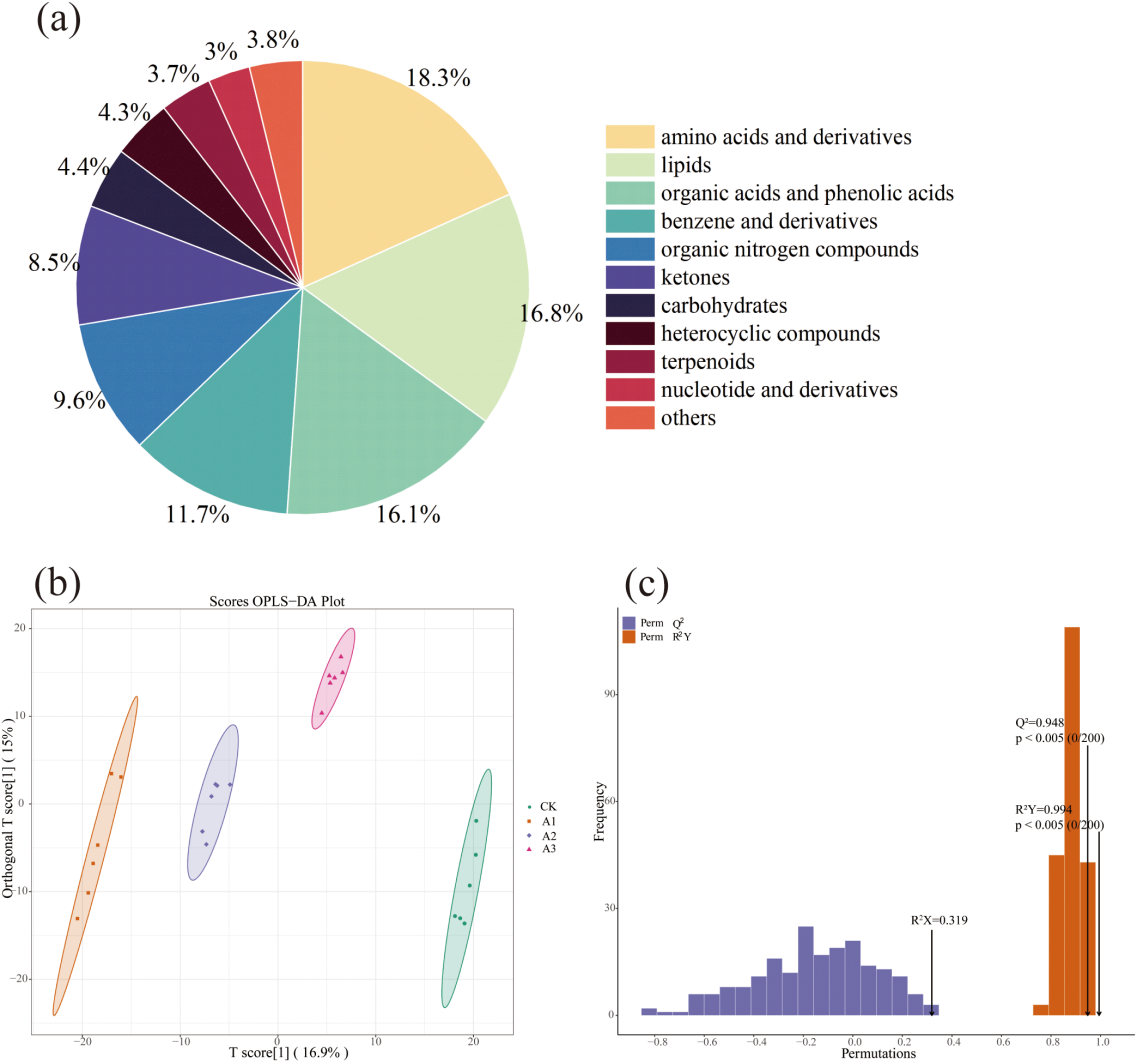
Metabonomic analysis of soil in *P. edulis*–*G. lucidum* agroforestry models a with different cultivation years. **(A)** Chemical classification of all identified metabolites. **(B)** OPLS-DA analysis of metabolites between different treatments. **(C)** Cross-validation model of OPLS-DA.

Using LC-MS untargeted metabolomics method, a total of 1218 metabolites were detected in the soil and classified into 11 chemical classes. This included amino acids and derivatives (18.3%), lipids (16.8%), organic acids and phenolic acids (16.1%), benzene and derivatives (11.7%), organic nitrogen compounds (9.6%), ketones (8.5%), carbohydrates (4.4%), heterocyclic compounds (4.3%), terpenoids (3.7%), nucleotide and derivatives (3.0%) and others (3.8%) (**Figure 6A**). Differences within and between the control and treatment groups were analyzed using the supervised discriminant analysis statistical method OPLS-DA model to determine the effects of cultivation year on soil metabolites (**Figure 6B**). There were differences between the four treatments. The OPLS-DA model indicated that the model was good and could be used to screen for DMs (R^2^Y = 0.994, Q^2^Y = 0.948) (**Figure 6C**), which differed among three cultivation years treatments and control groups. Correlation analysis was performed for all soil metabolisms among all comparison pairs (**Supplementary Figure 2**). Different years of cultivation had different effects on soil metabolism, as measured by the metabolites.

**Figure 7:**
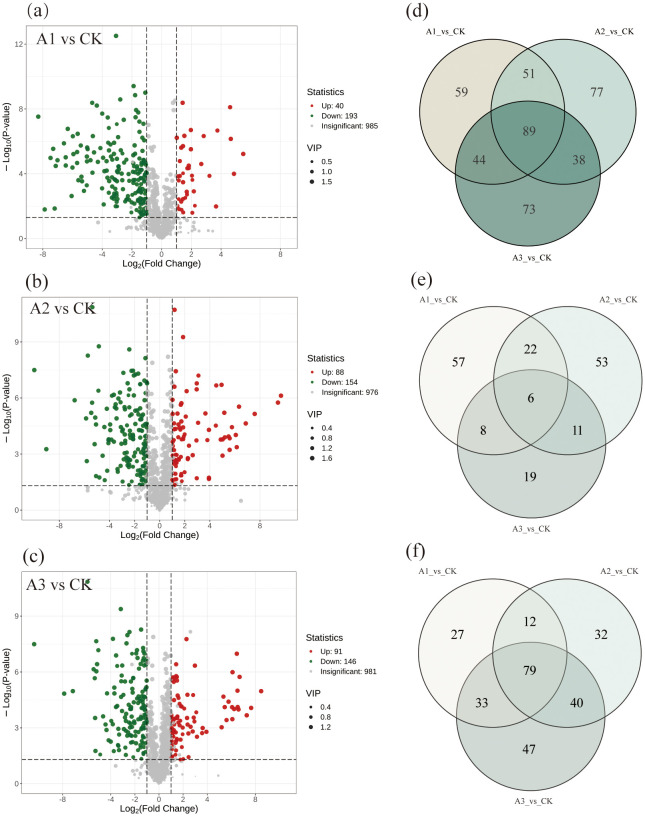
Different metabolites analysis of soil in *P. edulis*–*G. lucidum* agroforestry models along a chronosequence. **(A–C)** Soil metabolomics analysis of different in *P. edulis*–*G. lucidum* agroforestry systems with different cultivation years treatments vs CK. **(D)** Venn diagram of differentially abundant metabolites (DAMs) number across different cultivation years treatments vs CK. **(E)** Venn diagram for upregulated differentially abundant metabolites (DAMs) across different cultivation years compared to control (CK). **(F)** Venn diagram for downward differentially abundant metabolites (DAMs) across different cultivation years compared to control (CK).

**Figure 8:**
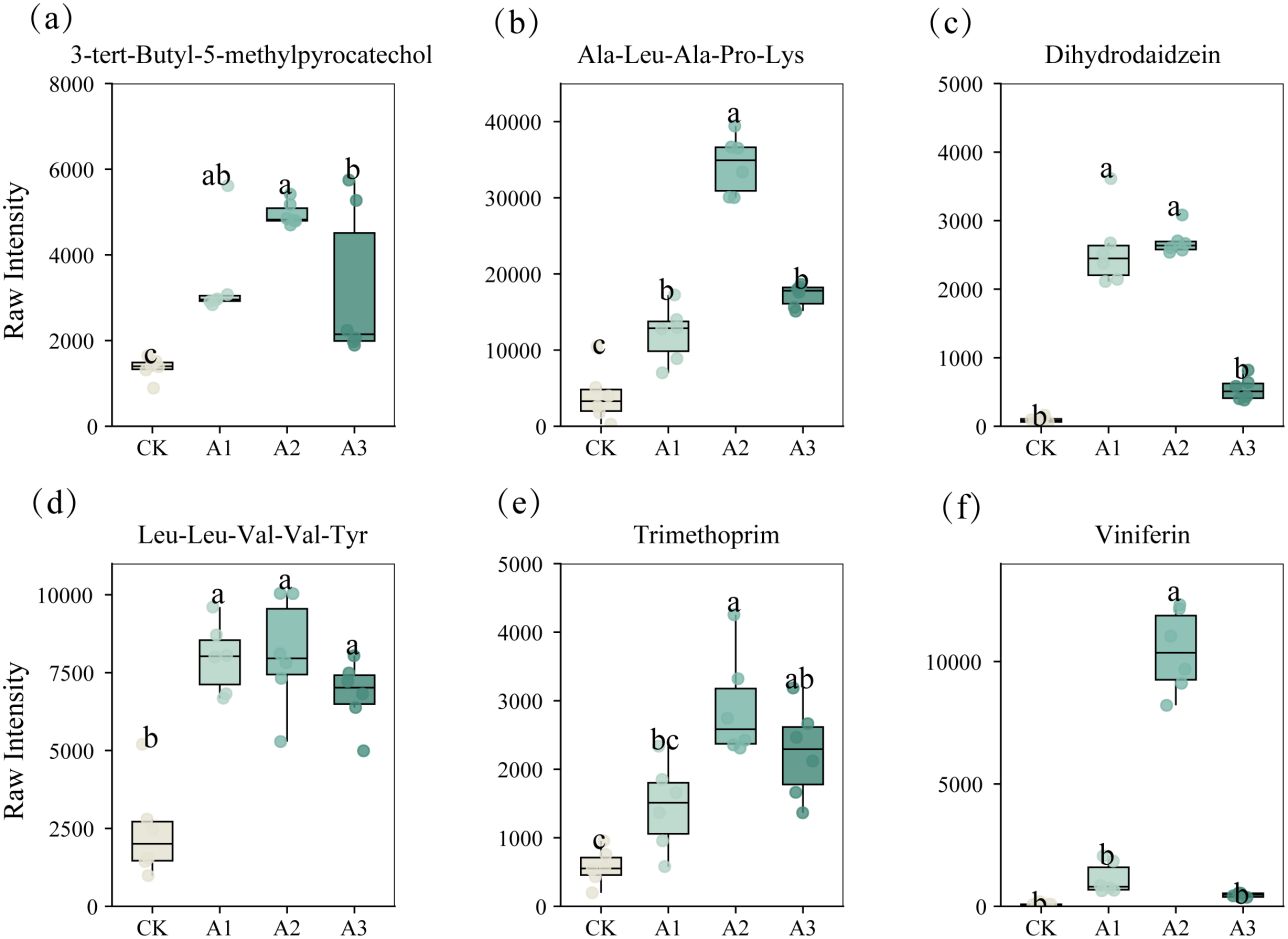
Content changes of common upregulated DAMs in *P. edulis–G. lucidum* agroforestry systems with different cultivation years treatments vs CK. **(A–F)** Raw intensity of 3-tert-Butyl-5-methylpyrocatechol, Ala-Leu-Ala-Pro-Lys, Dihydrodaidzein, Leu-Leu-Val-Val-Tyr, Trimethoprim, Viniferin.

Sample analysis showed that the two groups had biological replicates within each group, and there were significant differences between the groups. Therefore, the metabolites of the two groups can be screened according to the screening criteria: VIP ≥ 1, FC (fold change) ≥ 2, and FC ≤ 0.5. The DMs were screened using volcano plots (**Figures 7A–C**). Compared to CK, A1, A2, and A3 contained 233 (40 up, 193 down), 242 (88 up, 154 down), and 237 (91 up, 146 down) DMs, respectively. As shown in the Venn diagram of differentially abundant metabolites (**Figures 7D–F**), the unique DMs of A1, A2, and A3 compared with those of the CK were 59, 77, and 73, respectively, and the common DMs were 89. The common up DMs of A1, A2, and A3 compared with CK were six (3-tert-Butyl-5-methylpyrocatechol, Ala-Leu-Ala-Pro-Lys, Dihydrodaidzein, Leu-Leu-Val-Val-Tyr, Trimethoprim, Viniferin). Meanwhile, that of common down DMs was 79 (**Figures 8A–F**).

### 3.7 Correlations between soil carbon components and soil properties

**Figure 9:**
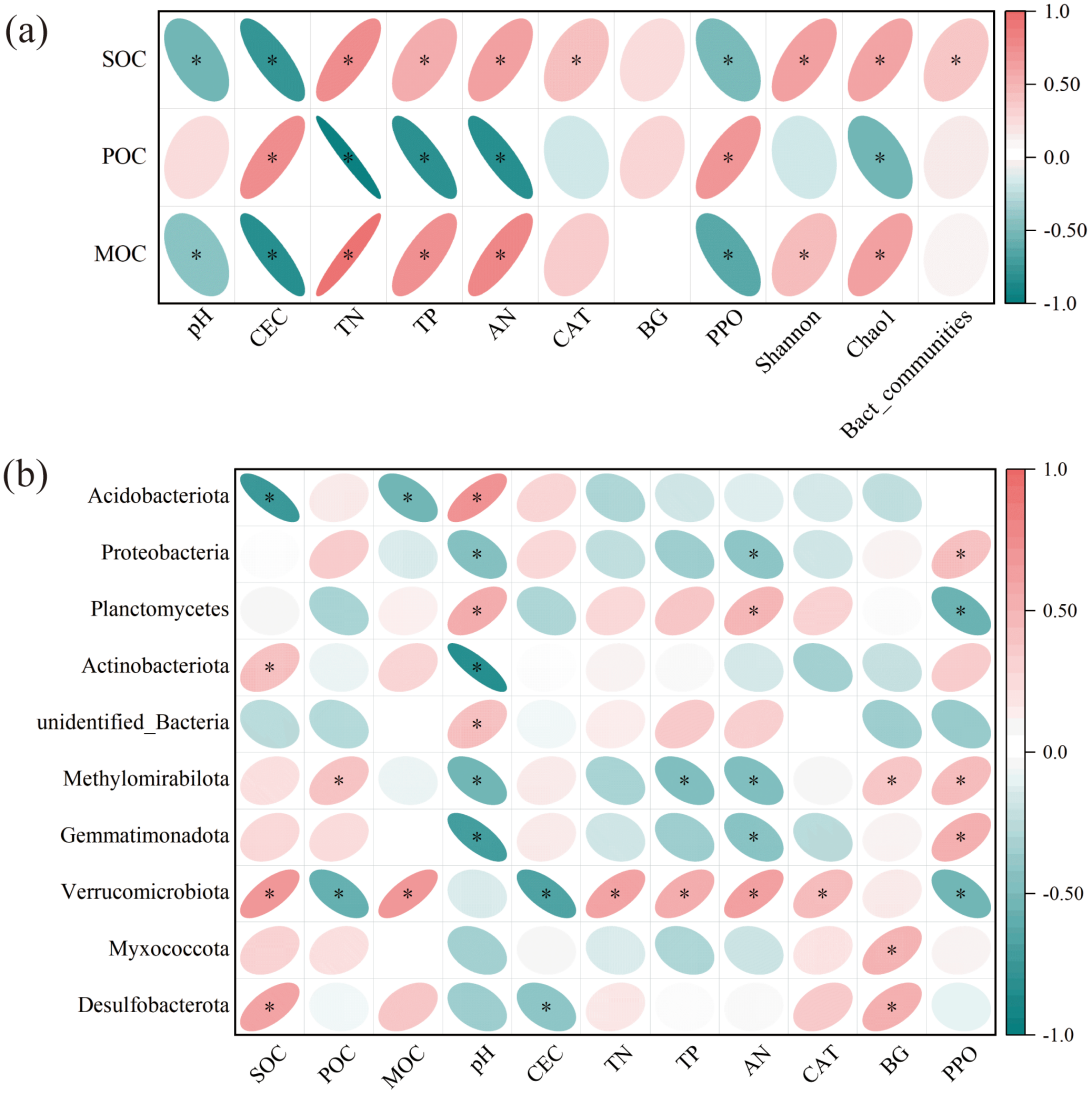
Correlation analysis among soil organic carbon components, basic properties, enzyme activities, and bacterial community compositions (phylum level) in *P. edulis*–*G. lucidum* agroforestry models with different cultivation years **(A)** Pearson correlation among soil carbon components, properties, enzyme activities and bacteria diversity. **(B)** Pearson correlation among soil bacteria relative abundance, soil carbon components, properties, and enzyme activities. *p<0.05.

Pearson’s correlation analysis was conducted to examine the relationships between soil organic carbon components, soil physicochemical properties, and enzyme activities in *P. edulis*–*G. lucidum* agroforestry models with different cultivation durations (**Figure 9A**). SOC, POC, and MOC were significantly correlated with multiple environmental factors (*p* < 0.05). SOC was significantly negatively correlated with pH, CEC, and PPO (*p* < 0.05) and positively correlated with TN, TP, AN, CAT, Shannon index, Chao1 index, and bacterial community composition (*p* < 0.05). MOC was significantly negatively correlated with pH, CEC, and PPO (*p* < 0.05) and significantly positively correlated with TN, TP, AN, Shannon index, and Chao1 index (*p* < 0.05). In contrast, POC was significantly negatively correlated with TN, TP, AN, and the Chao1 index (*p* < 0.05) but positively correlated with CEC and PPO (*p* < 0.05).

Pearson’s correlation analysis showed that the relative abundance of bacterial phyla was closely associated with SOC components, physicochemical properties, and enzyme activities (**Figure 9B**). Acidobacteriota showed significant negative correlations with SOC and MOC and a significant positive correlation with pH (*p* < 0.05). Proteobacteria were significantly negatively correlated with pH and AN but positively correlated with PPO (*p* < 0.05). Planctomycetes exhibited a significant negative correlation with PPO, and a significant positive correlation with pH and AN (*p* < 0.05). The relative abundance of Verrucomicrobiota was most strongly negatively correlated with POC and positively correlated with SOC and MOC (*p* < 0.05).

### 3.8 Correlations between soil carbon components, metabolites and bacterial communities

**Figure 10:**
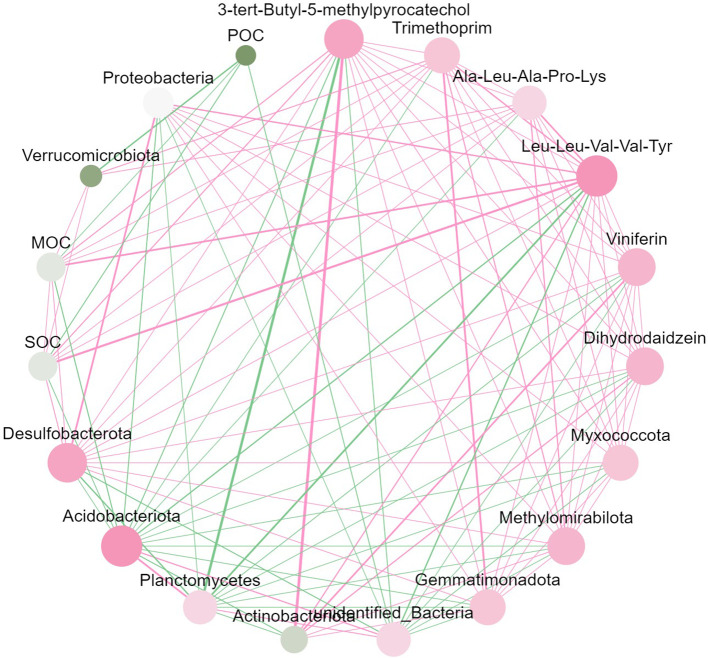
Spearman correlation network diagram of soil upregulated DMs with organic carbon components and bacterial communities. Pink and green arrows indicate significantly positive and negative relationships, respectively. Values on arrows represent standardized path coefficients.

A co-occurrence network was constructed between the differentially upregulated metabolites in the treatment groups, compared with CK, SOC components, and microbial phyla (**Figure 10**). These DMs were significantly positively correlated with SOC, POC, Verrucomicrobiota, and Actinobacteriota, and significantly negatively correlated with Acidobacteriota (*p* < 0.05). Most bacterial phyla, including Desulfobacterota, Acidobacteriota, and Myxococcota, display synchronized abundance dynamics with a broad set of metabolites such as 3-tert-butyl-5-methylpyrocatechol, Leu-Leu-Val-Val-Tyr, and Viniferin. SOC and SOC were significantly positively correlated with amino acids and their derivatives (Leu-Leu-Val-Val-Tyr and Ala-Leu-Ala-Pro-Lys), benzene and its derivatives (3-tert-butyl-5-methylpyrocatechol), organic acids, and phenolic acids (trimethoprim).

**Figure 11:**
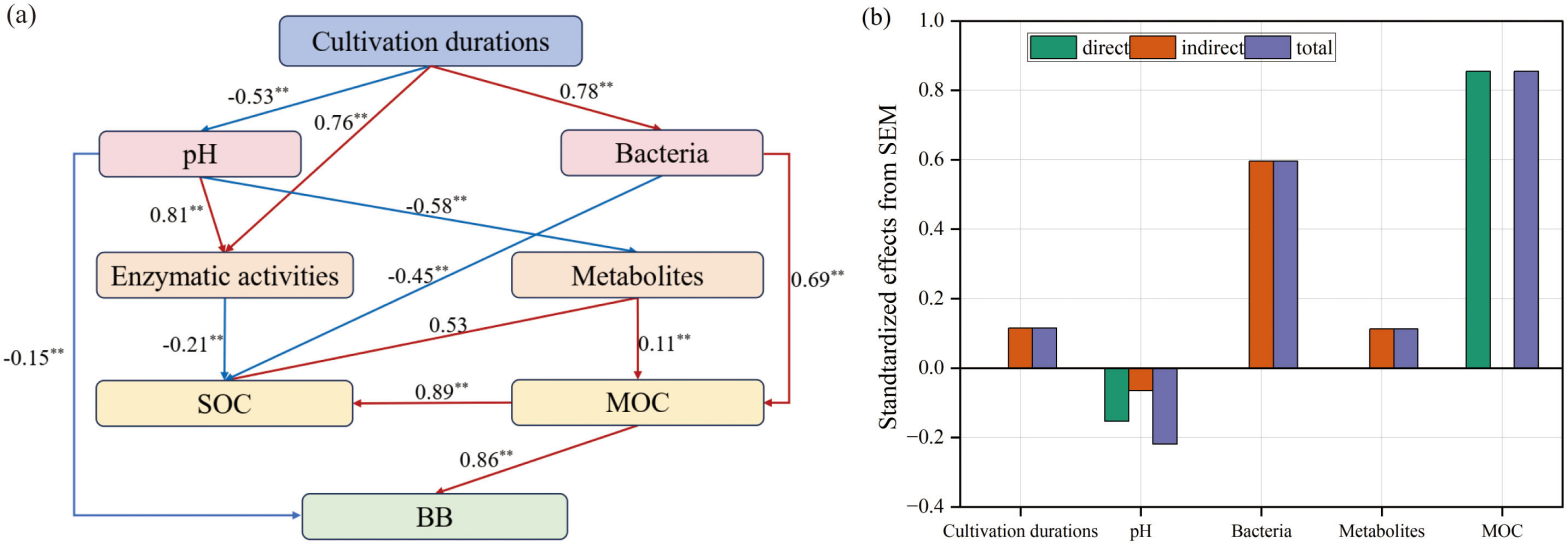
**(A)** Partial least squares structural equation modeling (PLS-SEM) analysis performed to evaluate pathways of cultivation durations influence on the soil properties, bacteria (Verrucomicrobiota, Actinobacteriota), enzymatic activities (CAT, BG), metabolites (Ala-Leu-Ala-Pro-Lys, Leu-Leu-Val-Val-Tyr), carbon components (SOC, MOC) and Moso bamboo aboveground biomass (BB). Red and blue arrows indicate significantly positive and negative relationships, respectively, while the indicated values on the arrows represent standardized path coefficients. (GOF = 0.609, *p < 0.05, **p < 0.01). **(B)** Standardized total effects of Cultivation durations, pH, bacteria, metabolites, and MOC.

SEM analysis showed that the cultivation duration had direct and indirect effects on soil pH, bacteria, enzymatic activities, metabolites, MOC, SOC and BB (bamboo aboveground biomass) (GFI = 0.609, **Figure 11**). Cultivation duration had indirect effects on MOC by negatively affecting pH (path coefficient = -0.53) and metabolites (path coefficient = -0.58, Leu-Leu-Val-Val-Tyr and Ala-Leu-Ala-Pro-Lys). Cultivation duration had indirect effects on SOC by negatively affecting pH (path coefficient = -0.53) and positively affecting enzymatic activities (path coefficient = 0.81). Planting duration promotes the bamboo aboveground biomass (BB) by positively driving shifts in soil bacterial communities (Verrucomicrobiota and Actinobacteriota) and MOC.

## 4 Discussion

### 4.1 Impacts of different cultivation durations on soil basic properties and organic carbon components in *P. edulis–G. lucidum* agroforestry systems

*G. lucidum* cultivation durations significantly altered the bamboo biomass, soil properties and organic carbon components. *G. lucidum* cultivation significantly reduced soil pH and CEC, and soil acidification became more pronounced with increasing years of cultivation (**Table 1**). This pattern is largely consistent with previous studies. This suggests that *G. lucidum* continuously modulates the rhizosphere chemical environment through hyphal exudation, microbial community restructuring, and enhanced organic matter decomposition and turnover. This diminishes the soil buffering capacity and influences cation adsorption and retention by soil colloids (Ji et al., 2024).

In terms of soil nutrients, understory cultivation of *G. lucidum* and other edible fungi generally increases soil C, N, and P pools (Cherubin et al., 2019; Liu et al., 2025). Our results are consistent with these findings, demonstrating that *G. lucidum* cultivation significantly elevated the soil total TN, TP, and AN content in Moso bamboo plantations. This nutrient enrichment can be largely attributed to the continuous input of organic substrates during *G. lucidum* cultivation, which mainly consisted of wood chips and bamboo residues. The decomposition of plant-derived materials replenishes the soil with abundant organic matter and essential nutrients, facilitating the overall accumulation of soil nutrient pools (Ge et al., 2025). Despite the increases in TN, TP, and AN, *G. lucidum* cultivation decreased soil available phosphorus (AP). In bamboo–fungus agroforestry systems, increased C and N inputs often stimulate bamboo growth, leading to higher numbers of new culms and shoots (Liu et al., 2022). Subtropical regions, where Moso bamboo forests are widely distributed, are generally characterized by P limitations (Tang et al., 2016; Zeng et al., 2024). Under such conditions, enhanced bamboo productivity may substantially increase plant P uptake, resulting in the continuous depletion of available soil P pools. Therefore, the observed decline in AP likely reflects an imbalance between the increased P demand associated with *G. lucidum*–induced bamboo growth and the relatively insufficient replenishment of soil AP (Guan et al., 2017; Wu et al., 2018).

In addition to N and P enrichment, *G. lucidum* cultivation significantly increased the SOC content in Moso bamboo plantations (**Figure 1**). This finding is consistent with most studies on forest–mushroom agroforestry systems, which have reported enhanced SOC accumulation following the introduction of edible or medicinal fungi (Pan et al., 2025; Ye et al., 2026). To further elucidate the effects of *G. lucidum* cultivation on soil organic carbon pools in Moso bamboo forests, we divided SOC into POC and MOC, two components with fundamentally different physicochemical properties, to better assess soil C stability and turnover (Lavallee et al., 2020).

Compared with the non-cultivated control, *G. lucidum* cultivation significantly increased both SOC and MOC content. Both components exhibited a continuous increase with increasing cultivation duration. *G. lucidum* cultivation substantially promotes SOC accumulation in Moso bamboo soils, favoring the formation of more stable C pools. Long-term SOC accumulation is not primarily derived from the direct buildup of plant residues, but rather depends on the microbial processing of organic substrates and the subsequent formation of MOC (Six et al., 2007a; Cotrufo et al., 2019). The sustained increase in MOC observed suggests that *G. lucidum* cultivation may enhance microbially mediated C transformation processes, increasing the stabilization and long-term retention of SOC. In the third year of *G. lucidum* cultivation (A3), the POC content declined significantly, whereas SOC and MOC continued to increase. This pattern indicates pronounced restructuring of the SOC pool with prolonged *G. lucidum* cultivation, whereby a portion of the labile organic C originally present as particulate components was increasingly decomposed or transformed and subsequently transferred into more stable C pools. Lavallee et al. (2020) reported that a decline in the relative contribution of POC often represents a transition toward carbon pool saturation or stabilization, rather than an SOC loss. Therefore, the substantial reduction in POC observed in the third year of cultivation likely reflects an accelerated shift from fast-to slow-cycling C components in the *P. edulis*–*G. lucidum* system (Lugato et al., 2021).

As a large wood-decaying mushroom, *G. lucidum* may play a critical role in this process through extensive hyphal growth and metabolic activity. Previous studies have highlighted the indispensable role of fungi and associated microbial communities in SOC stabilization, with fungal metabolites and microbial necromasses being the major sources of MOC (Kallenbach et al., 2016; Liang et al., 2017). By promoting the decomposition of complex organic compounds such as lignin and cellulose and enhancing microbial metabolic efficiency, *G. lucidum* may accelerate the transformation of POC and facilitate the incorporation of microbially derived C into MOC. This likely explains the continued accumulation of MOC concomitant with the decline in POC during the later cultivation stages.

### 4.2 Impacts of different cultivation durations on soil enzyme activities

*G. lucidum* cultivation significantly altered the activity of C-cycle-related enzymes in the soils of Moso bamboo plantations and different enzymes exhibited pronounced stage-dependent responses to cultivation duration. The introduction of *G. lucidum* influences soil C turnover pathways and stabilization mechanisms by regulating microbial functional processes. CAT activity was significantly higher in the middle and later stages of *G. lucidum* cultivation (A2 and A3) than in CK. This suggests that prolonged cultivation enhanced the microbial metabolic intensity and the oxidative stress regulatory capacity (Burns et al., 2013). As a typical wood-decaying fungus, *G. lucidum* may increase soil metabolic activity through extensive hyphal growth and its regulatory effects on the microbial community structure, sustaining elevated CAT activity levels. In contrast, PPO and BG displayed an initial increase, followed by a decrease, with increasing cultivation time. PPO activity increased significantly after one year of cultivation. This indicated an enhanced oxidative decomposition potential for aromatic compounds such as lignin and polyphenols. However, with prolonged cultivation, the PPO activity gradually declined and was significantly lower than that of the control in the third year (A3). This suggests a progressive depletion of readily oxidizable aromatic substrates and stabilization of some of the decomposition products through microbial processing and association with soil minerals (Six et al., 2007b; Cotrufo et al., 2015). Similarly, the BG activity peaked in the second year of *G. lucidum* cultivation (A2) and declined significantly in the third year (A3). Labile carbon availability was the highest during the mid-stage of cultivation, supporting the maximal microbial decomposition of easily accessible carbon substrates. In contrast, in the later stages, the depletion of labile carbon reduces microbial investment in hydrolytic enzymes (Sinsabaugh et al., 2008). This shift in enzyme activity corresponds to the observed decrease in POC and continued accumulation of MOC in the third year, further suggesting that *G. lucidum* cultivation promotes the transition of soil organic C from fast-cycling components to more stable pools after a certain cultivation period (Hansen et al., 2024).

### 4.3 Impacts of different cultivation durations on soil bacterial community structures

The soil bacterial community structure forms a fundamental biological basis for soil organic carbon transformation and nutrient cycling. *G. lucidum* cultivation significantly altered soil bacterial diversity and community composition in Moso bamboo plantations, with this effect intensifying as cultivation duration increased. These results align with previous findings that understory cultivation, such as fungal or crop introduction, typically reshapes soil microbial communities by altering carbon inputs and rhizosphere conditions (Dollinger and Jose, 2018; Cardinael et al., 2020; Dou et al., 2025). *G. lucidum* cultivation significantly increased soil bacterial richness and diversity (**Figure 3**). The ASV numbers in treatments A2 and A3 were higher than those in treatments CK and A1. Meanwhile, the Shannon and Chao1 indices generally increased initially and then stabilized. These results suggest that the middle and late stages of *G. lucidum* cultivation provide favorable habitats for diverse bacterial taxa. Hyphal proliferation and elevated litter inputs in understory cultivation systems increase organic substrate availability, increasing soil bacterial diversity (Nuralykyzy et al., 2025; Ye et al., 2026). Increased bacterial diversity is generally beneficial for improving the functional redundancy and stability of soil ecosystems, supporting complex organic matter transformations and nutrient cycling (Maron et al., 2018; Zhang et al., 2023).

*G. lucidum* cultivation significantly alters the relative abundance of soil bacterial communities at the phylum level. Acidobacteriota and Planctomycetota, which dominated the control, were significantly reduced under *G. lucidum* cultivation. Meanwhile, the relative abundances of Proteobacteria, Actinobacteriota, and Verrucomicrobiota increased. As oligotrophic taxa adapt to nutrient-poor conditions with low carbon-use efficiency, the decreased abundance of Acidobacteriota implies improved soil nutrient availability and reduced selection of oligotrophic bacteria (Fierer et al., 2007). Correspondingly, the enhanced relative abundance of copiotrophic or facultative copiotrophic groups, such as Proteobacteria and Actinobacteriota, reflects elevated labile carbon availability and greater soil microbial metabolic capacity (Zhou et al., 2016).

Different bacterial taxa responded differently to *G. lucidum* cultivation duration. Proteobacteria peaked in the second year (A2) and decreased slightly in the third year (A3). Meanwhile, Verrucomicrobiota and Planctomycetota increased significantly in A3. Proteobacteria often dominates under labile carbon-rich conditions, whereas Verrucomicrobiota and some Planctomycetota lineages are better adapted to low substrate availability or complex organic matter (Bergmann et al., 2011; Faria et al., 2018). Therefore, the successional shift in the bacterial community composition with cultivation duration likely reflects a transition in the soil system from a labile carbon-driven stage to a stable carbon-maintenance stage.

### 4.4 Impacts of different cultivation durations on soil metabolites

Soil metabolites mainly originate from plant roots and microorganisms. Here, amino acids and derivatives, lipids, organic acids, phenolic acids, benzene derivatives, and organic nitrogen compounds were the primary metabolites in the soil, all of which were related to microbial or plant growth regulation (Liu et al., 2023; Li et al., 2024). Intercropping can create a certain soil metabolic environment (Chen et al., 2024; Brown et al., 2024). Phenolic acid metabolite formation is associated with the ligninolytic ability of fungi. As a white-rot fungus, *G. lucidum* secretes lignin-degrading enzymes during its metabolic process. This decomposes bamboo litter to produce a large number of phenolic secondary metabolites. These substances are signaling molecules for *G. lucidum* growth and are key regulators of soil microorganisms. The high enrichment of peptide nodes such as Ala-Leu-Ala-Pro-Lys and Leu-Leu-Val-Val-Tyr reflected an accelerated soil nitrogen cycle after intercropping. Soluble nitrogen released via *G. lucidum* mycelial metabolism enhances microbial use of amino acid-derived carbon sources. This supports previous observations that intercropping elevates soil hydrolyzable N and ammonium N. Flavonoid metabolites, including viniferin and dihydrodaidzein, which exhibit both antimicrobial and growth-promoting activities, help regulate soil microbial community structure and mitigate continuous cropping obstacles (Ye et al., 2026).

### 4.5 Relationships among bamboo biomass, soil carbon components, metabolism and bacterial community

Microorganisms mediate soil organic matter decomposition and their carbon use regulates soil organic C turnover (Xue et al., 2024). Soil metabolites reflect microbial responses to soil nutrients (Li et al., 2019) and are byproducts of energy-intensive chemical transformations driven by microbial communities (Xue et al., 2024). Here, some key microbial phyla such as Actinobacteriota showed strong positive correlations with soil metabolites. This suggests that intercropping with *G. lucidum* may create a more favorable living environment for these beneficial microorganisms (Ye et al., 2024). The specific metabolites induced by treatments such as intercropping with *G. lucidum* are correlated with the SOC and POC contents and exhibit synergistic changes with beneficial microbial phyla such as Verrucomicrobiota and Actinobacteriota. This promotes the accumulation of soil C pools. The positive correlations between small-molecule peptides, such as Leu-Leu-Val-Val-Tyr, and phenolic acid compounds, including 3-tert-butyl-5-methylpyrocatechol, and SOC suggest that they may form key precursors or stabilizing factors for organic C formation. Most metabolites, including peptides and phenolic acids, showed significant positive correlations with Proteobacteria and Acidobacteriota. This indicated that nitrogen-containing substances produced by *G. lucidum* metabolism provide C and N sources for Proteobacteria, accelerating their proliferation. Proteobacteria further decompose metabolites, releasing available nutrients, forming a cyclic process of metabolite supply–bacterial proliferation–nutrient release (Liu et al., 2021). The significant negative correlation between Actinobacteriota and specific metabolites, including 3-tert-Butyl-5-methylpyrocatechol, may be attributed to Actinobacteriota secreting enzymes to degrade phenolic acid inhibitors while using them as carbon sources, avoiding the inhibitory effects of metabolite accumulation (Větrovsky et al., 2014; Bourguignon et al., 2016). The results showed that planting durations influenced the formation of MOC by positively altering soil bacterial Verrucomicrobia, Actinobacteriota. In our study, agroforestry significantly increased the Moso bamboo biomass by 30.2%–40.2%, this indicates that intercropping with *G. lucidum* promotes the growth of bamboo. MAOC serves as an important carrier of soil nutrients such as N and P, and its stable existence signifies long-term soil fertility and health. Our SEM analysis showed planting duration promotes the bamboo biomass by positively driving shifts in soil bacterial communities and MOC. These results demonstrate that microorganisms convert organic carbon into forms that can tightly bind with minerals through their metabolic activities, thereby enabling long-term stabilization in soil and promoting the growth of Moso bamboo.

## 5 Conclusions

Our study highlighted that agroforestry management improved soil organic C and bamboo aboveground productivity. Compared with pure bamboo forests, cultivation of *G. lucidum* significantly increased the SOC and MOC content, soil catalase activity, enhanced bacterial community diversity and richness, and enriched the relative abundances of Proteobacteria, Actinobacteriota, and Verrucomicrobiota. Six significantly upregulated differential metabolites were shared between different cultivation durations and belonged to the categories of amino acids and their derivatives, organic acids, and benzene and its derivatives. Further analysis revealed that planting duration influenced the formation of MOC by positively altering the soil bacterial Actinobacteriota, Verrucomicrobia, and soil amino acids and derivatives. Collectively, these findings advance our understanding of the coupled mechanisms linking agroforestry practices, microbial communities, metabolites, and soil C dynamics, while offering theoretical guidance for the sustainable management, ecological restoration, and C sink enhancement of managed Moso bamboo plantations.

## Data Availability

The original contributions presented in the study are included in the article/**Supplementary Material**. Further inquiries can be directed to the corresponding author.
