# Supplementary material for: Effects of cultivation duration on soil organic carbon components, bacterial communities, metabolites and bamboo growth in the Phyllostachys edulis– Ganoderma lucidum agroforestry systems
Source: Front Plant Sci. 2026 Jul 30;17:1917212. doi: 10.3389/fpls.2026.1917212 (PMC13469285; doi:10.3389/fpls.2026.1917212)
Supplement: Supplementary file 1 [file DataSheet1.docx]

(a)

(b)

Fig.S1 Beta diversity indices, LefSe analysis of soil bacteria in *P. edulis*–*G. lucidum* agroforestry models

(a)

(b)

(c)

Fig.S2 Heatmap of DMs species across different cultivation years treatments vs CK
